# The Dentist’s Right to Practice Medicine

**Published:** 1889-11

**Authors:** C. M. Wright


### The Dentist's Right to Practice Medicine.

BY PROF. C. M. WRIGHT.

Read before the Seventh Ohio District Dental Society, September 17, 1889.

The question has occurred frequently to my mind, "How much of general medicine has a dentist the right, legally or honestly, to practice?"

In the cities and towns of America, the reader of signs will find on the doors themselves, or on the door posts, or upon hanging signs of tin or wood over the doors of certain houses, situated in various parts of these cities and towns, announcements like the following: "John Doe, Surgeon Dentist," "James Roe, Dentist," "William Poe, D.D.S., Dentist," "Samuel Hoe, M.D., D.D S., Dentist," etc. These signs indicate to the passing public that the gentlemen whose names appear on the signs are dentists, or in other words practitioners of the dental art. They are appealing through these door plates and signs to the public's teeth. "Oh ye, who have toothache! Oh ye, who don't want to have toothache! Oh ye, who have no teeth! come in here and be treated. John Doe will file and polish, scrape and chisel, probe and plug your diseased teeth. James Roe will extract your old broken down roots and crowns of useless teeth, and replace them with handsome clean ones of beautiful china. Here we all are—dentists, we are surgeon dentists, D.D.S. dentists, M.D., D.D. S. dentists." These signs certainly cry out in this way to the passing public, even if the accomplished and graceful "operator" is above the necessity of receiving the general public, and only desires the visits of the favored wealthy, or cultivated units of the public. If a man in walking along the streets after luncheon, suddenly bites down upon a bit of sand or a stem in his plug-tobacco, and strikes a pulp and twangs a nerve in the pulp of a tooth, that man acts like an Indian on the war-path seeking a foe--and he is hunting for a sign--just such a sign as we have been describing. He runs by many a house with the simple name Dr. Blank on the sign. What he wants—and he wants it right away--is a *dentist.* Yet that man has other symptoms that are systemic, or general in character.

Observe the spasmodic twitchings, the *risus sardonicus,* that might indicate to the general practitioner--tetanus; observe the peculiar contractions of the muscles of the extremities and the action of the joints, pointing to a phase of *locomotor ataxy*; see the flushed face with sweat pouring from the brow and running in streams down the cheeks; feel the bounding pulse; listen to the throbbing heart--no, this is not toothache, but some profound agitation of the great nerve centres, affecting seriously the organs of the circulation of the blood and the motor nerves of the muscles, and of the secretory glands of the skin. Why don't that man go to a doctor for systemic treatment?

He finds a dentist's "shop," and the man behind the counter picks out the impinging sand or the tobacco stem, applies a local antalgic and in a short time all the violent general pathological symptoms fade away. Possibly the dentist administers an anæsthetic, suspends his customer over the grave by a hair for fifteen seconds, during which short interval an expert would say, the man has the *grand mal* fit of an epilepsy, extracts the tooth and sends the subject out of his office in a joyful mood.

All this is legitimate business and accords with the general idea—the accepted relations of the dentist to the public. So far, and in a number of similar acts, or operations on the part of the dentist, there can hardly be a question, legal or otherwise, raised against the dentist, (unless the patient should not recover from the anæsthetic—but this I don't want to discuss, just now).

There are other matters, however, that come up in a dentist's office. Recognizing as he does that the teeth are parts or a part of the organism, intimately connected with the great circulatory and nerve centers, and consequently affected more or less by distant irritations in the organism—dependent upon the condition of general nutrition—suffering temporarily or permanently from deficient, or mal-nutrition of the organism—liable to local disorders which affect other near or remote organs or functions of organs—he, the dentist, might, I say, feel disposed to administer medicines, foods, etc., etc., with the hope of bettering the general health, and with it the teeth themselves. Now the question comes up—Has the man with the sign "dentist" hanging at his door the right to treat the whole organism? Has he that right even if he has D.D.S., Dentist, or M.D., D.D.S., Dentist, on his sign? Does the public expect it? Should the public pay for it? Or could the dentist collect a fee for medical treatment?

Has not the regular practitioner a right to object to this infringement of his rights, this trespassing upon his territory? I have often listened to the complaint of the dentist against a neighboring physician for extracting teeth, treating alveolar abscesses, etc., etc., and yet this *specialist* makes no bones of running all over the grounds of the neighboring physician. He would feel very much insulted, if perchance, the physician should say, "See here, dentist, why did you prescribe iron and bark, or phosphate of lime, or what not to Miss So and So? She needed preparatory treatment before she could take the iron at all." Or "dentist, you should have known something of Mrs. So and So's idyosyncracy before you prescribed sulph. morph. or antipyrine in such doses as you did."

Why didn't the dentist send his patient to the family doctor? Why didn't the dentist, if he regularly intends to practice medicine, call upon his patient as a doctor and watch the results of his medicine, as the doctor certainly has a right to do?

Gentlemen, I am not here to accuse any body of doing any thing that is not honest or legitimate. I am not here to cast reflections upon the status of the dentist. I am only a dentist, and have no sympathy whatever with the man who is ashamed of the occupation of his life, and yet who lives by it and brings up his sons and daughters to despise their father's occupation (or profession). Unfortunately, there are too many of us who occupy this senseless position. It is pitiable, indeed, for a dentist, even an M.D. dentist, to despise his own rank and swell himself up to such gaseous proportions that he begins to imagine that possibly somebody may rank him with the common doctor. The frog can not, by inflating himself with wind become an ox, let him then try only to be the biggest frog in his own puddle.

I have desired only to ask questions that may be asked by higher authority some day. Questions which it might be well for us to answer to our own satisfaction for ourselves. Personally, I love to play at doctor, to lay aside, for the moment, the character of the hard-working, exact science, must do-something-useful-that-will-wear,-or-not-get-paid kind of man that the dentist must be, and putting on my nose-glasses, taking the wrist of my patient in one hand and my watch in the other, feel and count the gentle thub-thub of the pulse. Then I like to look wise, and say, "Let me see your tongue, Madame"—"Hum, "um!" "Have you had a chill?" "Hum, "um." Then what can be more inspiring of confidence, more elevating to one's personal dignity than at this stage of the proceeding to take out a little pad of druggists' paper and carefully indite in Latin one of the dozen prescriptions I have so carefully committed to memory.

Oh, I know the pleasures of the practice of general medicine, brothers, but, is this our business? That is the question I wanted to ask to-day. I have here an article clipped from a medical journal that presents a similar question in a light that may help to illustrate our way. It is a judge's opinion on the use of the title *Homœpathist*, and as I say I like it, I should much rather play with the materia medica than with the knives, and saws, and forceps of the surgeon and the gynecologist, or than with the dental engine and the forceps of the dentist, if I could see only people who would not die on my hands, and if I could confine my practice to the hours of the day. In other words, if I could play at doctoring without having the gnawing responsibility of health and life itself on my mind.

